# Complete resolution of a giant multilocular prostatic cystadenoma following androgen deprivation therapy: an illustrative case report

**DOI:** 10.1093/omcr/omab053

**Published:** 2021-07-21

**Authors:** Nikita Sushentsev, Yvonne Rimmer, Tristan Barrett

**Affiliations:** 1 Department of Radiology, Addenbrooke’s Hospital and University of Cambridge, Cambridge CB2 0QQ, UK; 2 Department of Oncology, Addenbrooke’s Hospital and University of Cambridge, Cambridge CB2 0QQ, UK

## Abstract

Giant multilocular prostatic cystadenoma (GMPC) is a rare benign pelvic mass for which complete surgical resection is an accepted treatment of choice. This report presents the first case of complete resolution of GMPC following a 3-year course of luteinising hormone-releasing hormone agonist alongside external beam radiotherapy for the concurrent treatment of unfavorable intermediate-risk prostate cancer. In addition to illustrating the imaging features of the effect of androgen deprivation therapy (ADT) and radiotherapy on GMPC regression, this case provides evidence for considering ADT as an alternative, noninvasive GMPC treatment option in patients in whom surgical treatment is either contraindicated or can be made less invasive by reducing the size of GMPC prior to its removal.

## INTRODUCTION

Giant multilocular prostatic cystadenoma (GMPC) is a rare benign tumor with <30 cases reported in literature [1] since its first description by Maluf *et al*. [2] in 1991. GMPC often manifests with obstructive urinary symptoms, urinary retention or pelvic pain. Complete surgical resection is the treatment of choice, however, not all patients are suitable for a surgical intervention, thereby presenting a case for alternative, noninvasive GMPC treatment options. In this report, we present the first case of a patient in whom GMPC was concurrently diagnosed with an unfavorable intermediate-risk prostate cancer (PCa), with both lesions showing complete resolution following a 3-year course of luteinizing hormone-releasing hormone (LHRH) agonist in combination with external beam radiation therapy (EBRT).

## CASE REPORT

**Figure 1:**
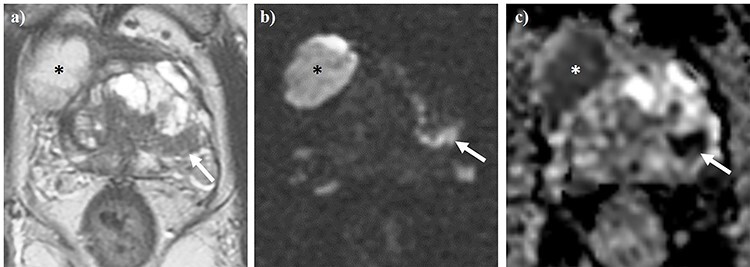
Axial T2WI (a) and diffusion-weighted imaging (b), alongside apparent diffusion coefficient (ADC) mapping (c) of the pelvis. The white arrow denotes a PI-RADS 5 lesion located in the left peripheral zone. Asterisks demonstrate one of the locules of a concurrent giant multilocular prostatic cystadenoma.

**Figure 2:**
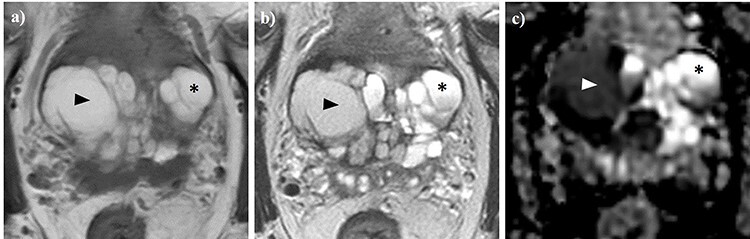
Axial T1WI (a) and T2WI (b), alongside apparent diffusion coefficient (ADC) mapping (c) of the pelvis. A giant multilocular prostatic cystadenoma is observed. Locules on the right (arrow heads) demonstrate high T1 (a) and intermediate T2 (b) signal intensity, as well as low ADC values (c), which is consistent with blood products. In contrast, locules on the left (asterisks) exhibit high T1 signal, higher T2 signal intensity (b) and ADC values (b), likely relating to proteinaceous fluid.

**Figure 3:**
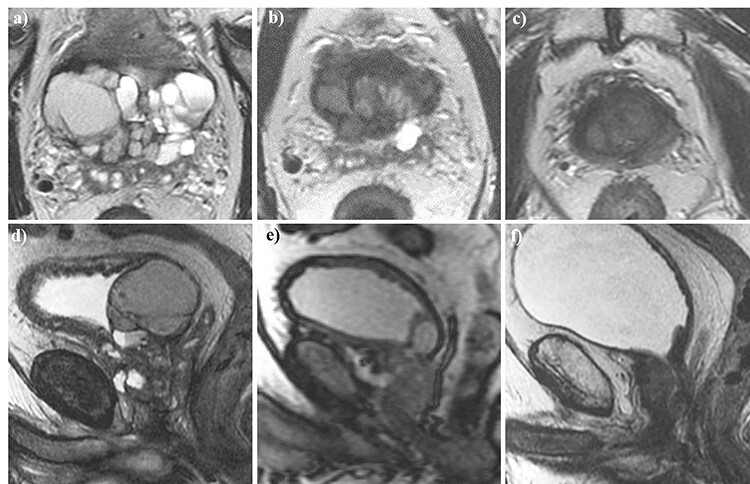
Axial (a–c) and sagittal (d–f) T2W magnetic resonance imaging of the pelvis performed at baseline (a, d), 3 months (b, e), and 3.5 years (c, f) following the commencement of androgen deprivation therapy (ADT). The giant multilocular prostatic cystadenoma observed at baseline showed a significant reduction after 3 months of ADT before resolving completely 3.5 years into treatment.

**Figure 4:**
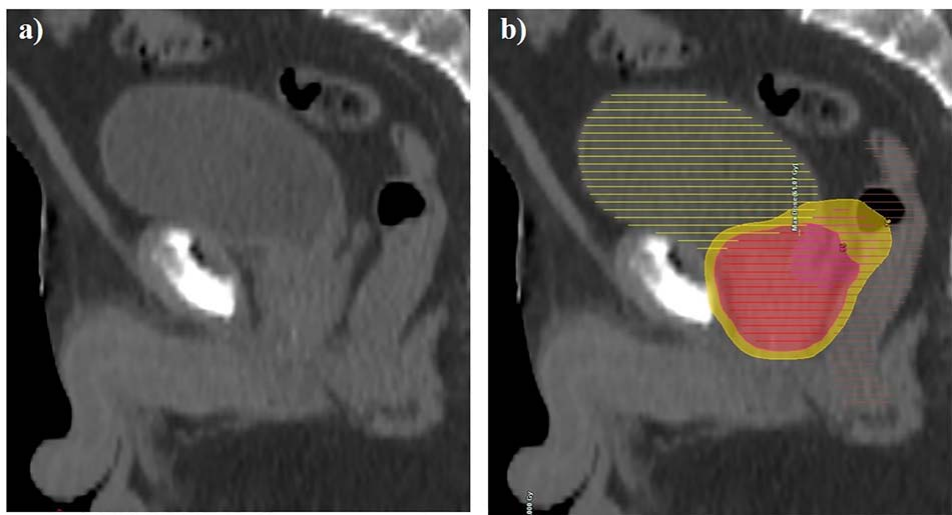
Sagittal external beam radiotherapy computed tomography planning scan performed at 6 months following the commencement of ADT (a–b). No suspicious mass protruding into the urinary bladder was convincingly visible on anatomical imaging (a). The patient received intensity-modulated radiation therapy using 60 Gy Prostate (red 95% isodose) and 48Gy seminal vesicles (yellow 95% isodose) delivered over 20 sessions with daily image guidance (b).

A 73-year-old male was referred to our center for transrectal ultrasound-guided prostate biopsy due to clinical suspicion for localized PCa with an elevated prostate-specific antigen (PSA) of 8.0 ng/ml. The biopsy revealed the presence of a Gleason score 4 + 3 = 7 mixed ductal and acinar carcinoma of the prostate in 3/3 cores in the left base, which was later correlated to a 17 × 7 × 15 mm (0.96 cm^3^) PI-RADS 5 lesion on staging multiparametric magnetic resonance imaging (mpMRI) of the prostate **(**Fig. 1**)**. mpMRI also demonstrated a multiloculated cystic lesion arising from the prostatic base and extending into the bladder, measuring 72 × 41 × 53 mm (81.9 cm^3^) **(**Fig. 2**)**. On the right side, the locules were of intermediate T2 signal with areas of restricted diffusion, likely relating to blood products. On the left side, the locules showed higher T2 signal with no restricted diffusion, and areas of low T1 or high T1 signal intensity, likely relating to either simple fluid or proteinaceous material, respectively. No imaging features of local invasion were observed, with the described radiological appearance being in keeping with GMPC. A subsequent flexible cystoscopy showed no invasion of the mass into the bladder, and no malignant cells were seen on diagnostic cytology. The patient was prescribed an LHRH agonist with a 3-month interval MRI demonstrating a significant reduction in size of GMPC, then measuring 43 × 29 × 40 mm (26.1 cm^3^) **(**Fig. 3**)**. The previously noted lesion in the left prostatic peripheral zone also appeared less prominent, measuring 11 × 4 × 9 mm (0.20 cm^3^). Following 6 months of ADT, the patient underwent hypofractionated EBRT with 60 Gy delivered over 20 treatment sessions. No MRI was performed immediately post-EBRT, however, the EBRT planning CT scan demonstrated even further reduction of GMPC, making it virtually undetectable **(**Fig. 4**)**. Following EBRT, the patient continued on LHRH agonist for a further 2 years with his PSA remaining < 0.04 ng/ml. A routine surveillance MRI scan, performed more than three and a half years after initial treatment, showed complete regression of both GMPC and the previously identified left peripheral zone lesion, with the final gland volume being 10.6 cm^3^  **(**Fig. 3**)**.

## DISCUSSION

To our knowledge, this is the first report of complete regression of a GMPC following a three-year course of androgen deprivation therapy (ADT) in combination with EBRT for organ-confined PCa. The 4-fold reduction in the GMPC volume reported after the initial 3-month ADT, alongside its further reduction seen on EBRT computed tomography (CT) planning scan, highlight the key role of castration in the observed phenomenon, likely driven by targeting the androgen receptor, which is known to be expressed on GMPC cells [1]. These findings are in line with the single published report of noninvasive treatment of recurrent unresectable GMPC with a gonadotropin-releasing hormone antagonist [3], which also demonstrated a positive result, with no further recurrence observed on continuous ADT. In the present report, interim EBRT could also have an add-on effect on GMPC resolution, e.g. by means of destroying its feeding vessel. Overall, these findings suggest that ADT is effective in treating both primary and recurrent GMPC, thereby serving as a promising alternative to invasive surgical treatment.

GMPC can be diagnosed at any age between 16 and 80 [1], with its presenting symptoms being similar to those of benign prostatic hyperplasia, including lower urinary symptoms, urinary retention, hematuria and elevated serum PSA levels. Radiologically, GMPCs present as large retroperitoneal masses located between the bladder and rectum, showing multilocular cavities and cysts of various sizes. T2-weighted and diffusion-weighted imaging may provide additional information on the presence and the nature of any GMPC soft tissue components [4]. Being aware of radiological features of GMPC, alongside other pelvic masses or congenital anomalies of the genitourinary tract, is of considerable clinical relevance given the increasing use of MRI for diagnosis, local staging and follow-up imaging of PCa, which may lead to a more frequent incidental detection of this rare condition [5, 6]. Additionally, to our knowledge, the concomitant diagnosis of GMPC and biopsy-proven PCa demonstrated in this report has not been presented previously.

As there are no pathognomonic histopathological signs of GMPC that could enable its confident diagnosis by biopsy [7], it is important to consider several differential diagnoses that include other lower male genitourinary tract cysts (Müllerian, prostatic, seminal vesicle and utricle), prostatic leiomyoma, lymphangioma, teratoma, liposarcoma, hydatid disease and phyllodes variant of atypical prostatic hyperplasia [1].

Given the size and possible adherence to surrounding organs, complete surgical excision of GMPC, which is required to prevent the disease recurrence [1, 2], often becomes a traumatic operation with major complications being bladder and rectum perforation, pelvic abscess, anal canal injury and rectal leak [1, 8]. As evidenced by this report, a short-term ADT course may be used as a preoperative volume-reducing step aimed at decreasing the invasiveness of GMPC surgical removal. In cases when radical treatment is contraindicated or can be avoided due to the mild nature of GMPC-related symptoms, strong consideration should be given to ADT as an alternative, noninvasive treatment option capable of achieving complete GMPC resolution.

## CONCLUSION

In conclusion, we reported the first case of complete regression of a GMPC observed on consecutive MRI scans following 3 years of ADT with an LHRH agonist. The reported observation warrants further attention to ADT as an alternative, noninvasive treatment option for patients with GMPC in whom complete surgical resection is either contraindicated or can be deferred.
